# A patient with familial Mediterranean fever mimicking diarrhea-dominant irritable bowel syndrome who successfully responded to treatment with colchicine: a case report

**DOI:** 10.1186/s13256-022-03446-z

**Published:** 2022-06-24

**Authors:** Shima Kumei, Masatomo Ishioh, Yuki Murakami, Katsuyoshi Ando, Tsukasa Nozu, Toshikatsu Okumura

**Affiliations:** 1 Department of General Medicine, Asahikawa Medical University, Asahikawa, Japan; 2 Division of Metabolism, Systemic Bioscience, Gastroenterology and Hematology/Oncology, Department of Medicine, Asahikawa Medical University, Asahikawa, Japan; 3 Department of Regional Medicine and Education, Asahikawa Medical University, Asahikawa, Japan

**Keywords:** Familial Mediterranean fever, Irritable bowel syndrome, Colchicine

## Abstract

**Background:**

Irritable bowel syndrome is a functional gastrointestinal disease. Visceral hypersensitivity is the most important pathophysiology in irritable bowel syndrome. Currently, diagnosis of irritable bowel syndrome is based on symptoms and exclusion of other organic diseases. Although the diagnosis of irritable bowel syndrome can be made based on the Rome IV criteria, one may speculate that complete exclusion of other organic diseases is not so easy, especially in cases uncontrolled with standard therapies.

**Case presentation:**

We present herein a case of familial Mediterranean fever in a young Japanese patient who had been suffering from an irritable bowel syndrome-like clinical course. A 25-year-old Japanese male had been diagnosed as having diarrhea-predominant irritable bowel syndrome 5 years earlier. Unfortunately, standard therapies failed to improve irritable bowel syndrome symptoms. After careful medical history-taking, we understood that he had also experienced periodic fever since 10 years ago. Although no mutation was identified in the Mediterranean fever gene, not only periodic fever but abdominal symptoms improved completely after colchicine administration. He was therefore diagnosed as having familial Mediterranean fever and that the abdominal symptoms may be related to the disease.

**Conclusions:**

Familial Mediterranean fever should be considered as a cause of irritable bowel syndrome-like symptoms.

## Background

Irritable bowel syndrome (IBS) is a functional gastrointestinal disease with a high population prevalence [1, 2]. Clinical symptoms of IBS include abdominal pain and stool irregularities, as well as other somatic, visceral, and psychiatric comorbidities [1, 2]. Currently, diagnosis of IBS is based on symptoms and exclusion of other organic diseases. Although a diagnosis of IBS can be made based on the Rome IV criteria [2], one may speculate whether complete exclusion of other organic diseases is not so easy, especially in cases uncontrolled with standard therapies.

Familial Mediterranean fever (FMF) is a genetic autoinflammatory disease characterized by recurrent fever with serosal inflammation [3]. FMF is considered to be rare in Japan, and the clinical features in Japanese tend to be milder than those observed in the Mediterranean population due to different genotypes of the Mediterranean fever (MEFV) gene [4, 5]. We present herein a case of FMF in a young Japanese patient who had been suffering from an IBS-like clinical course such as chronic severe diarrhea and abdominal pain. In clinical practice, we have experienced patients with an unknown cause of chronic diarrhea. When organic diseases are excluded by clinical tests or images including gastrointestinal endoscopy in patients with chronic diarrhea, we used to make a diagnosis of functional gastrointestinal diseases such as IBS. Based on the present case, we should consider FMF in the differential diagnosis of such patients with chronic diarrhea of unknown cause.

## Case report

A 25-year-old Japanese male complained of uncontrollable watery diarrhea over 10 times a day and abdominal pain since 5 years ago. Abdominal pain was mild lower abdominal pain relieving after defecation. There was no convincing aggravating factor for the abdominal pain. He had never had any health problems in the past. There was no particular social, environmental, or family history. His occupation was civil servant. He had never smoked and had drunk socially. Medical examination at the first visit revealed blood pressure of 120/72 mmHg, pulse rate, of 72 times/minutes (regular), and body temperature of 36.2 °C. There were no obvious abdominal and neurological findings, including abdominal tenderness.

He had received esophagogastroduodenoscopy, colonoscopy, and capsule endoscopy in addition to urine and blood examinations, and abdominal imaging such as abdominal computed tomography and magnetic resonance imaging (MRI). However, no abnormal findings had been detected, and he was diagnosed with diarrhea-dominant irritable bowel syndrome (D-IBS) based on the Rome IV criteria. He then received oral medicines including loperamide (1.0 mg/day) for 1 month, albumin tannate (3.0 g/day) for 1 month, or ramosetron hydrochloride (5 μg/day) for 1 month without improvement of his abdominal symptoms prior to his first visit to our department.

**Fig. 1:**
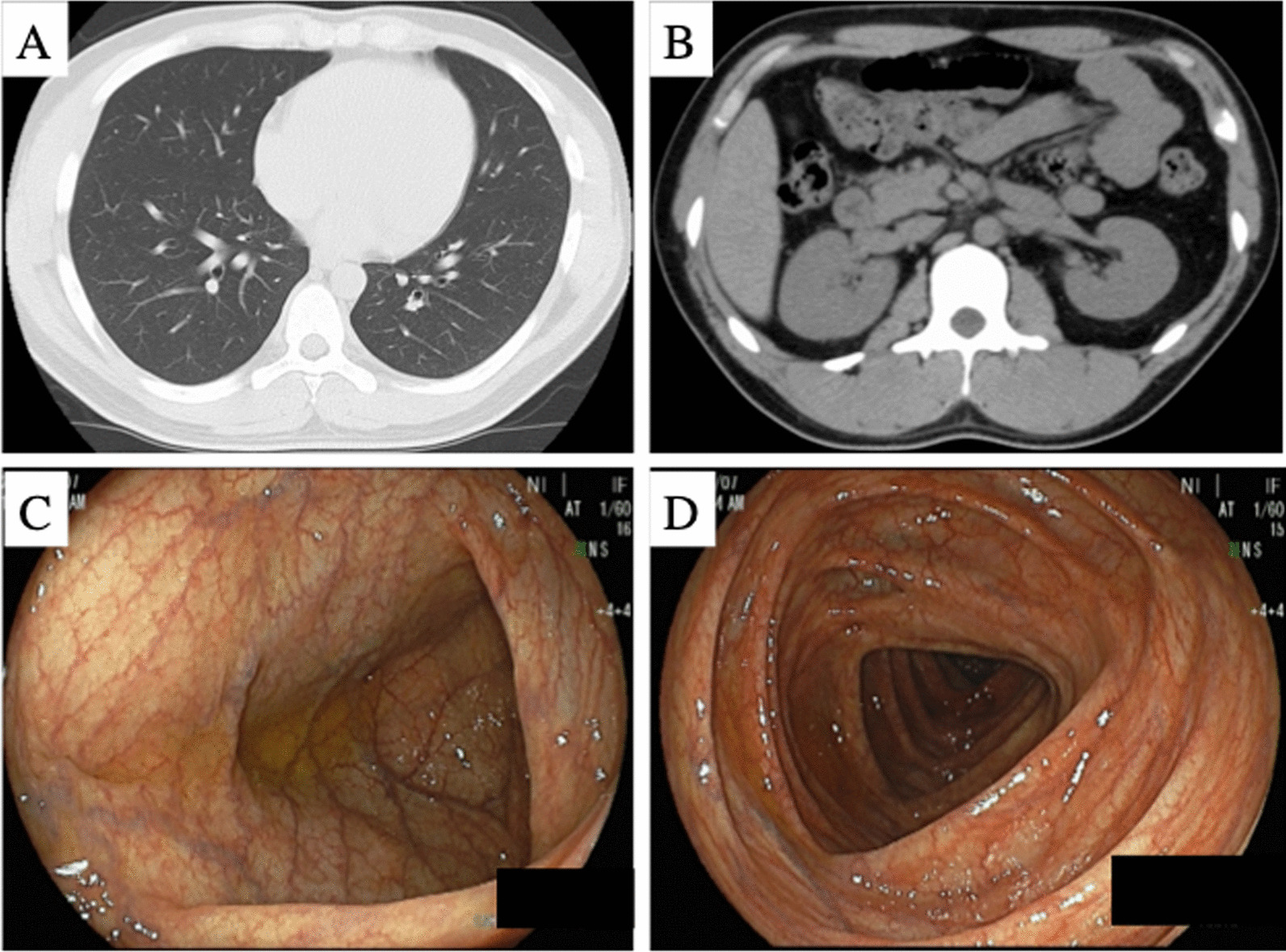
Clinical images of this case: **A** chest CT scan, **B** abdominal CT scan, **C** colonoscopy for sigmoid colon, and **D** colonoscopy for ascending colon

**Fig. 2:**
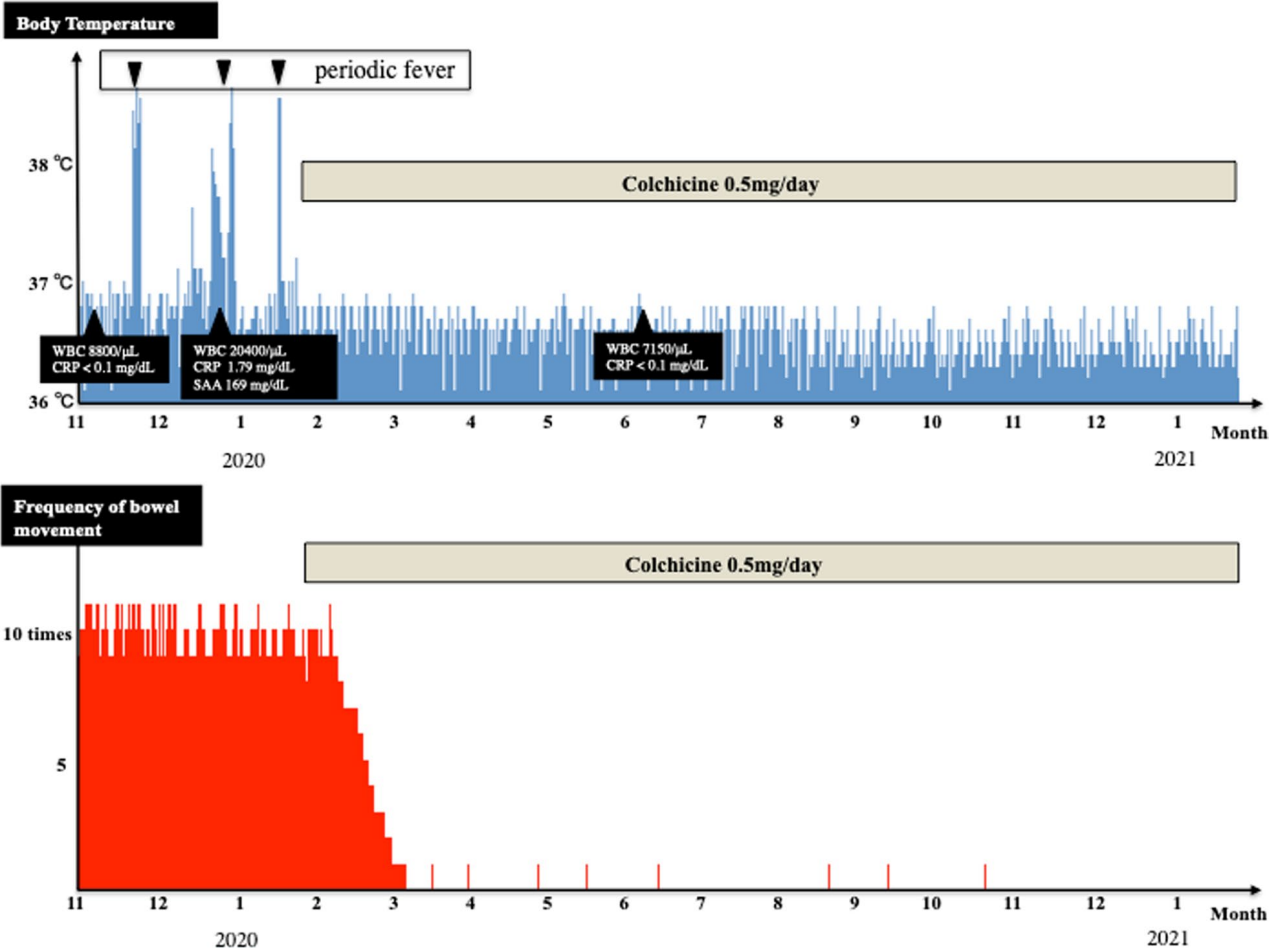
Time course of clinical characteristics in this case

He had been introduced to our department because of uncontrollable D-IBS for a long time. Laboratory testing including TSH was normal (Table 1). Whole-body computed tomography reveled no abnormality (Fig. 1A, B). To rule out collagenous colitis, infectious diseases, and celiac disease, colonoscopy including terminal ileum, random biopsies, and culture test of intestinal juice were performed and no macroscopic, pathological abnormality such as collagen band or possible disease-causing bacteria was observed (Fig. 1C, D). After careful medical history-taking, we understood that he had been experienced periodic fever since 10 years ago. Fever is characterized by recurrent (approximately once a month) and self-limited (a couple of days) fever. Figure 2 shows the time course of clinical characteristics. Blood examination on the day of fever revealed high white blood cell (WBC) count (20,400/μL), C-reactive protein (CRP) (1.79 mg/dL), and amyloid A protein (169 mg/dL) without anemia. However, during the interval between febrile attacks, his body temperature was normal and the laboratory data of inflammatory reaction were at normal levels. These clinical characteristics suggested a suspicion of familial Mediterranean fever (FMF), although we did not confirm that diarrhea and abdominal pain were associated with FMF. After 12 months since the start of colchicine administration at 0.5 mg/day, the patient has been free of fever. As shown in Fig. 2, not only periodic fever but also abdominal symptoms such as severe diarrhea improved completely after colchicine administration. All exons of the MEFV gene in the patient were sequenced, and no mutation was identified. Based on these findings, we concluded a diagnosis of FMF according to the criteria in Japan [4] in spite of the lack of gene mutation, and that abdominal symptoms may be related to the disease.

**Table 1:** Laboratory data at first visit to our department (normal body temperature)

WBC	8800	/µl
Neu	67.4	%
Lymp	25.5	%
Mono	4.4	%
Eos	2.2	%
Baso	0.5	%
RBC	536 × 10^4^	/µl
Hb	16.	g/dl
Plt	25.5 × 10^4^	/µl
TP	7.2	g/dl
Alb	4.7	g/dl
T-Bil	0.4	mg/dl
ChE	434	IU/l
ALP	322	IU/l
AST	28	IU/l
ALT	48	IU/l
LDH	211	IU/l
γGTP	65	IU/l
CK	142	IU/l
AMY	86	IU/l
BUN	10.1	mg/dl
Cre	0.9	mg/dl
UA	6.7	mg/dl
Na	142	mEq/l
K	4.3	mEq/l
Cl	106	mEq/l
Ca	9.5	mEq/l
CRP	< 0.10	mg/dl
Fe	59	µg/dl
UIBC	319	µg/dl
TIBC	378	µg/dl
Ferritin	238.3	ng/dl
HbA1c	5.8	%
T-cho	236	mg/dl
TG	500	mg/dl
FT4	1.31	ng/dl
TSH	2.79	µIU/ml

WBC: white blood cell, RBC: red blood cell, Hb: Hemoglobin, Plt: platelet, TP: total protein, Alb: albumin, T-Bil: total bilirubin, ChE: cholinesterase, ALP: alkaline phosphatase, AST: aspartate aminotransferase, ALT: alanine aminotransferase, LDH: Lactate Dehydrogenase, γGTP: γ-glutamyl transpeptidase, CK: creatine kinase, AMY: amylase, BUN: blood urea nitrogen, Cre: creatinine, UA: uric acid, CRP: c-reactive protein, UIBC: unsaturated iron binding capacity, TIBC: total iron binding capacity, T-cho: total cholesterol, TG: triglyceride, FT4: free thyroxine, TSH: thyroid-stimulating hormone

## Discussion

To our knowledge, there are no reports on the relationship between chronic diarrhea and FMF. The present case suggests that FMF should be considered to be a cause of chronic diarrhea, especially when organic gastrointestinal diseases are excluded.

**Fig. 3:**
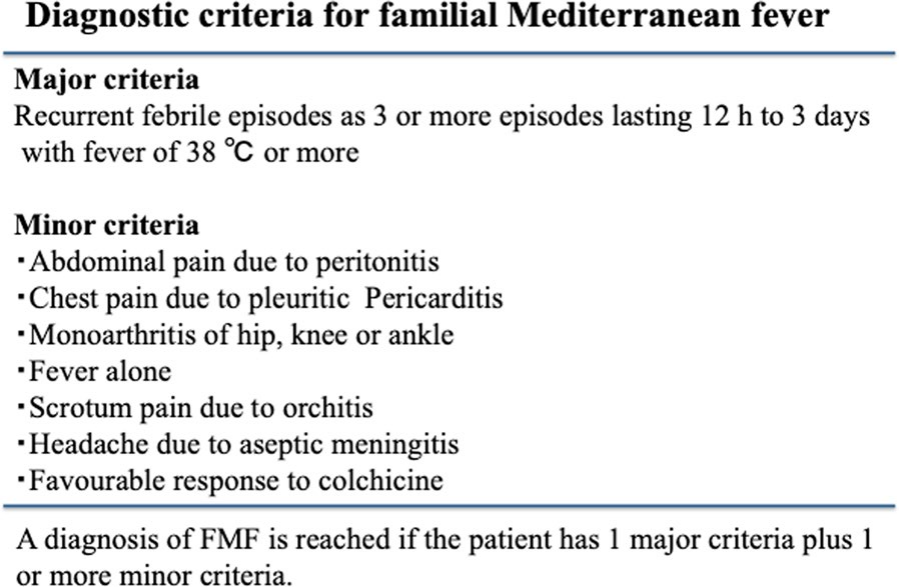
Diagnostic criteria for FMF in Japan [4]

FMF is characterized by self-limiting fever episodes, usually accompanied by serositis, arthralgia, and arthritis [3]. The diagnosis of FMF is made based on patient history, inflammatory markers, and genetic testing [6]. Several clinical diagnostic criteria sets have been proposed for diagnosis of FMF. The Tel Hashomer criteria, Livneh criteria, and Turkish pediatric criteria all rely on clinical symptoms, family history, and colchicine response [7–9]. In the diagnosis of Japanese patients with FMF, modified Tel Hashmer criteria (Fig. 3) have been suggested [4]. These include: recurrent febrile episodes (three or more episodes lasting 12 h to 3 days with fever ≥ 38 °C), and eight minor criteria as shown in Fig. 3. A diagnosis of FMF is determined if the patient exhibits the major criteria and one or more minor criteria. Differential diagnoses include infections, malignancy, and autoinflammatory diseases. In the present case, we diagnosed FMF because of recurrent febrile episodes and a favorable response to colchicine.

With regard to treatment for FMF, there are limited RCTs assessing interventions for patients with FMF in the most recent Cochrane Database [10]. Based on the evidence, daily colchicine may reduce the number of attacks. It has also been described that canakinumab probably reduces the number of attacks, and anakinra or canakinumab probably reduce CRP in colchicine-resistant participants. Thus, further research should be carried out to establish standard therapy for FMF.

With regard to a relationship between FMF and gastrointestinal disorders, accumulating evidence has suggested that some FMF patients exhibit intestinal mucosal lesions resembling inflammatory bowel diseases (IBD) [11, 12]. Uslu *et al*. [13] demonstrated that disease-causing *MEFV* mutations and the FMF disease rate were increased among patients with IBD. This increase was prominent among CD patients. Thus, FMF is considered to be a possible cause in patients with chronic intestinal mucosal lesions. However, in the present case, upper and lower gastrointestinal endoscopy detected no mucosal lesions in the gastrointestinal tract. These findings suggested that chronic diarrhea and abdominal pain came from functional but not organic gastrointestinal diseases such as IBD. Ekinci *et al*. examined a relationship between FMF and functional gastrointestinal disorders [14]. In their report on Turkish people, D-IBS was observed in 3 out of 103 FMF patients (2.9 %) and 2 out of 100 healthy subjects (2 %), respectively, suggesting that coexistence of FMF and D-IBS was not high in FMF when compared with healthy control. In other words, we suggest that D-IBS was observed incidentally in patients with FMF. In the present case, D-IBS developed from FMF rather than other causes incidentally because colchicine completely prevented not only typical FMF-induced febrile attacks but also severe diarrhea and abdominal pain.

The present case demonstrates that FMF can present IBS-like symptoms without intestinal mucosal lesions. In clinical practice, we do not usually rule in/out FMF when IBS is considered to be a highly possible diagnosis in patients with diarrhea and abdominal pain without any organic abnormality. FMF can be diagnosed even if mutation of MEFV gene is not detected. In Japan, MEFV gene mutation is not detected in approximately 5% of patients with FMF [15]. The present case suggests that patients with FMF may manifest gastrointestinal disease mimicking IBS, because colchicine potently improved not only periodic fever but also gastrointestinal symptoms such as diarrhea and abdominal pain.

## Conclusions

FMF should be considered as a cause of IBS-like symptoms, especially in patients diagnosed with IBS who do not respond to conventional IBS treatment.

## Data Availability

Data relevant to this case report are not available for public access because of patient privacy concerns but are available from the corresponding author on reasonable request.

## Abbreviations

IBS: Irritable bowel syndrome
FMF: Familial Mediterranean fever
MEFV: Mediterranean fever
D-IBS: Diarrhea-dominant irritable bowel syndrome
IBD: Inflammatory bowel diseases
